# Cytochrome P450 family 4 subfamily F member 2 (*CYP4F2*) rs1558139, rs2108622 polymorphisms and susceptibility to several cardiovascular and cerebrovascular diseases

**DOI:** 10.1186/s12872-018-0763-y

**Published:** 2018-02-09

**Authors:** Tao Zhang, Kuiying Yu, Xuhua Li

**Affiliations:** 1 First Department of Neurology, The First Hospital of Zibo, No.4 Emei Mountain Road, Boshan District, Zibo City, Shandong 255200 People’s Republic of China; 2 China Medical University Hospital of Boshan District, Zibo City, Shandong 255200 People’s Republic of China

**Keywords:** *CYP4F2*, Single nucleotide polymorphisms, Coronary artery disease, Hypertension, Ischemic stroke

## Abstract

**Background:**

Inconsistent conclusions have been reported for the genetic relationship between *CYP4F2* (Cytochrome P450 Family 4 Subfamily F Member 2) polymorphisms and the susceptibility to cardiovascular and cerebrovascular diseases.

**Methods:**

We performed a meta-analysis to assess the potential role of rs1558139 C/T and rs2108622 G/A polymorphisms of *CYP4F2* in the risks of cardiovascular and cerebrovascular diseases. The retrieval of four databases, including PubMed, Web of Science (WOS), China National Knowledge Infrastructure (CNKI) and WANFANG DATA, was conducted. Mantel-Haenszel statistics for association test, Cochran’s Q statistic, sensitivity analysis for heterogeneity assessment, and Begg’s/Egger’s tests for publication bias evaluation were performed under allele, homozygote, heterozygote, dominant, and recessive models, respectively.

**Results:**

A total of 597 articles were initially obtained by database searching, and twenty eligible articles were finally included. For rs1558139, a decreased risk of cardiovascular and cerebrovascular diseases was observed in the overall meta-analysis and in “hypertension”, “population-based” and “male” subgroups under models of T vs. C, CT vs. CC, and CT + TT vs. CC [all *P* values in association tests < 0.05, odds ratio (OR) < 1]. For rs2108622, a decreased coronary artery disease (CAD) risk was observed in the subgroup meta-analysis based on disease type under all genetic models (all *P* values in association tests < 0.05, OR< 1). Begg’s/Egger’s tests excluded the potential publication bias, while sensitivity analysis data supported the stability of the above results.

**Conclusion:**

C/T genotype of *CYP4AF2* rs1558139 may be linked to the decreased risk of hypertension in the male patients of Asian populations, while *CYP4F2* rs2108622 is likely associated with reduced susceptibility to CAD.

**Electronic supplementary material:**

The online version of this article (10.1186/s12872-018-0763-y) contains supplementary material, which is available to authorized users.

## Background

Single nucleotide change-induced nucleic acid sequence polymorphisms, namely, single nucleotide polymorphisms (SNPs), occur more frequently in the human genome, with an average of approximately one polymorphism per 1000 bases, and functional SNPs may help to guide population genetics research, as well as the mechanistic, epidemiologic or diagnostic study of some clinical genetic diseases [1, 2]. Emerging SNPs of different genes have been implicated in the complicated etiology or pathogenesis of several cardiovascular and cerebrovascular diseases [3–5]. For example, GWAS (genome-wide association studies) data show that some SNPs, such as rs1004467 of the *CYP17A1* (Cytochrome P450 Family 17 Subfamily A Member 1) gene or rs2681492 of *the ATP2B1* (ATPase Plasma Membrane Ca^2+^ Transporting 1) gene, are associated with susceptibility to blood pressure or essential hypertension [6, 7]. The aim of the present study was to investigate the potential role of the *CYP4F2* (Cytochrome P450 Family 4 Subfamily F Member 2) polymorphism in the risk of cardiovascular and cerebrovascular diseases, such as myocardial infarction (MI), coronary artery disease (CAD), ischemic stroke (IS), cerebral infarction (CI) and hypertension.

Human *CYP4F2* gene is located on chromosome 19 and contains 12 introns and 13 exons [8]. There are several common SNPs, such as rs3093100, rs3093105, rs3093166, rs1558139 and rs2108622 [8, 9]. The SNPs of rs3093100, rs3093105, rs3093166, and rs1558139 are located in the intron region of the *CYP4F2* gene, while rs2108622 (namely, V433 M, G1347A, and G20597A) is located in the exon region of this gene [8, 9]. CYP4F2 protein belongs to the human cytochrome P450 superfamily and functions as an essential enzyme for the production of 20-HETE (20-hydroxy eicosane arachidonic acid) [10, 11]. Increased 20-HETE levels are associated with vascular oxidative stress, endothelial dysfunction and high peripheral vascular resistance [10, 12]. As a bioactive eicosanoid and therapeutic intervention target, 20-HETE is involved in several vascular events, such as the modulation of blood pressure, renal function, cerebral blood flow and pulmonary circulation [10, 12].

Considering the conflicting conclusions, we quantitatively measured the genetic correlation between *CYP4F2* SNPs and the risk of cardiovascular and cerebrovascular diseases via meta-analysis based on the publicly published data. To our knowledge, one meta-analysis of hypertension [13] and another meta-analysis of ischemic stroke [14] were previously reported for *CYP4F2* rs2108622. However, no meta-analysis for the other SNPs of the *CYP4F2* gene have been reported. Additionally, there is no meta-analysis regarding the role of *CYP4F2* rs2108622 in the risk other cardiovascular and cerebrovascular diseases.

## Methods

### Database searching

In accordance with the methodology of “Preferred Reporting Items for Systematic Reviews and Meta-Analyses (PRISMA)” [15], we performed the retrieval of four electronic databases, including PubMed, Web of Science (WOS), China National Knowledge Infrastructure (CNKI) and WANFANG DATA, up to Jan 2018. Considering the limitation of publication space, the searching terms for each database are listed in supplemental online Additional file 1: Table S1.

### Selection criteria

After screening, eligible case-control studies provided the sufficient genotype frequency data of *CYP4F2* gene in each case/control group. The following exclusion criteria were considered: a) animal or cell experiments; b) other genes/diseases or non-SNP gene variants; c) meeting abstract; d) meta-analysis; e) review or letter; f) undetected variants; g) the absence of genotype frequency data; h) duplicated articles; and i) not in HWE (Hardy-Weinberg-Equilibrium).

### Basic information

We extracted the basic information from the eligible articles, namely, first author, year, ethnicity, genotyping assay, SNPs, genotype frequency, disease type, gender, mean age, study number, sample size of the case/control, X^2^ and *P* values of HWE, and source of control. The paper quality was assessed by the Newcastle-Ottawa Scale (NOS) system [16], which indicates poor quality when the NOS score is less than five.

### Statistical analysis

Based on Mantel-Haenszel statistics, the value of OR (odds ratio), 95% CIs (confidence intervals) and *P* value of association test was generated under the allele, homozygote, heterozygote, dominant, and recessive models. A fixed-effect model was used when the *P* value of heterogeneity from Cochran’s Q statistic was larger than 0.05 or I^2^ less than 50.0%. Subgroup meta-analyses by ethnicity (Caucasian/Asian), disease type (hypertension/CAD/IS/CI/MI), source of control (population/hospital -based), and gender (male/female), were also conducted through Mantel-Haenszel statistics. For publication bias assessment, Begg’s/Egger’s tests were performed. Stata/SE software version 12.0 (StataCorp LP,College Station, TX 77845 USA) was used for the above statistical analyses.

## Results

### Eligible case-control studies

**Fig. 1:**
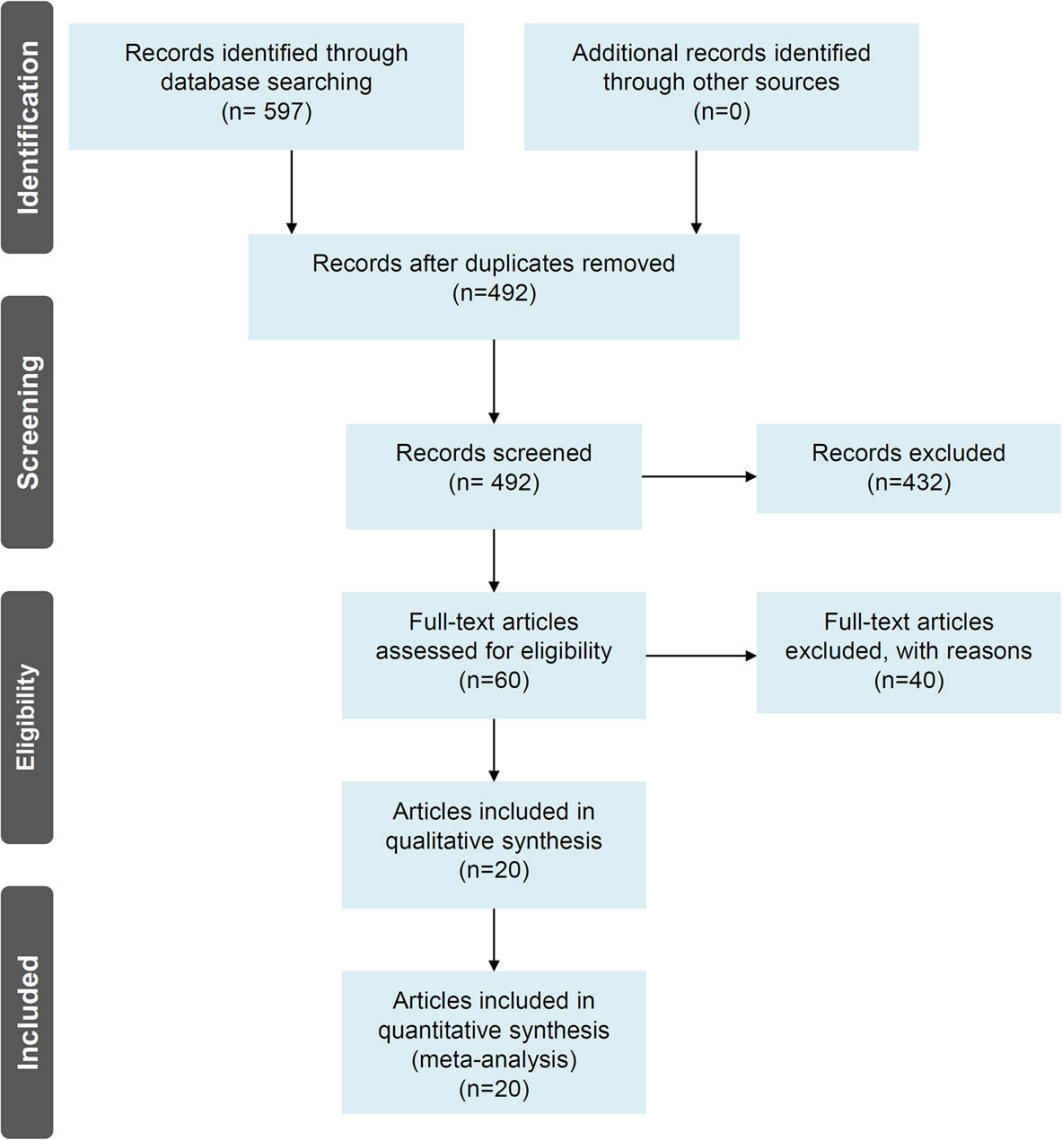
Flow diagram of the eligible case-control studies identified

Eligible case-control studies were obtained following the flow diagram of Fig. 1. We first obtained 597 articles from database searching, namely, PubMed with 128 articles, WOS with 369 articles, CNKI with 36 articles, and WANFANG DATA with 65 articles. Next, we removed 105 articles with duplicated data and 432 articles did not met the inclusion criteria. A total of 60 full-text articles were then assessed for eligibility. We further removed 40 articles for undetected variants or the absence of genotype frequency data. Finally, 20 articles [8, 9, 17–34] were included in the present meta-analysis. The basic information and genotype frequencies of eligible case-control studies for the present meta-analysis are provided in Additional file 1: Tables S2-S5. Two polymorphisms within the *CYP4F2* gene (rs1558139 C/T and rs2108622 G/A) were analyzed. The NOS system data suggested that no included studies showed poor quality, as all NOS scores were larger than five (Additional file 1: Table S3). Moreover, all the genotype distributions of controls adhered to Hardy-Weinberg Equilibrium (Additional file 1: Table S4-S5).

### The rs1558139 C/T polymorphism

**Fig. 2:**
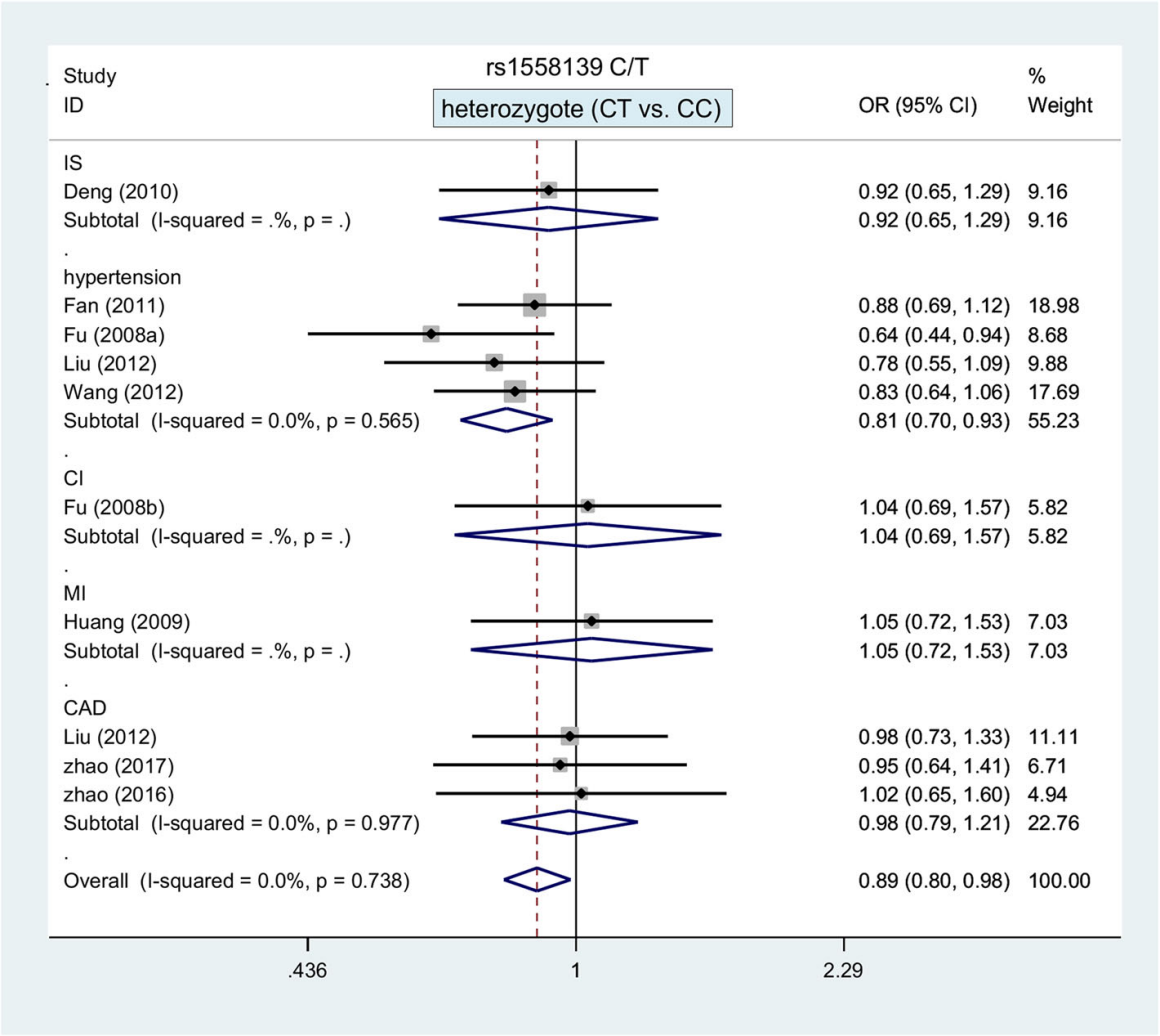
Subgroup analysis by disease type of the association between the *CYP4AF2* rs1558139 polymorphism and the risk of cardiovascular and cerebrovascular diseases under the heterozygote model

We first conducted a meta-analysis to analyze the association between *CYP4AF2* rs1558139 C/T and risk of cardiovascular and cerebrovascular diseases. In Table 1, ten case-control studies with 3462 cases and 3547 controls in an Asian population were enrolled [8, 18, 19, 21, 22, 25, 27, 33, 34]. Overall meta-analyses and subgroup analyses were conducted under allele (G vs. A), homozygote (GG vs. AA), heterozygote (AG vs. AA), dominant (AG + GG vs. AA), and recessive (GG vs. AA+AG) genetic models. There was no high degree of heterogeneity in any of the meta-analyses (Table 1, I^2^ value =0.0%, *P* value of heterogeneity > 0.05), and fixed-effect model was applied. In pooled data from overall meta-analysis (Table 1), a decreased risk of cardiovascular and cerebrovascular diseases was observed between case and control groups (T vs. C, *P* value in association tests = 0.021, OR = 0.92; CT vs. CC, *P* = 0.024, OR = 0.89; CT + TT vs. CC, *P* = 0.012, OR = 0.88). Moreover, similar results were observed under allele, heterozygote and dominant genetic models in “hypertension” and “population-based” subgroups (Table 1, all *P* value in association tests < 0.05, OR< 1). We also performed a subgroup analysis based on gender. As shown in Additional file 1: Table S6, a reduced hypertension risk was observed in the “male” subgroup under allele (*P* value in association tests < 0.001, OR = 0.79), homozygote (*P* = 0.009, OR = 0.68), heterozygote (*P* < 0.001, OR = 0.67) and dominant (*P* < 0.001, OR = 0.67) genetic models. Figure 2 shows the forest plot of the subgroup analysis by disease type under the heterozygote model, and Additional file 2: Figure S1 and Additional file 3: Figure S2 presents the data under allele and dominant models. Additional file 4: Figure S3 shows the forest plot of subgroup analysis by gender under the allele model. Thus, these results demonstrated that the C/T genotype of *CYP4AF2* rs1558139 might be associated with a reduced risk of hypertension in the male patients of Asian populations.

**Table 1:** Meta-analysis of *CYP4AF2* rs1558139 C/T polymorphism

Genetic models	Subgroup	Test of association			N	Sample size		Heterogeneity	
		OR (95% CIs)	z	*P* value		case	control	I^2^	*P* value
allele (T vs. C)	overall/Asian	0.92 (0.86, 0.99)	2.31	0.021	10	3462	3547	0.0%	0.931
	hypertension	0.89 (0.81, 0.98)	2.34	0.019	4	1852	1787	0.0%	0.768
	CAD	1.00 (0.87, 1.14)	0.05	0.963	3	883	914	0.0%	0.685
	population-based	0.91 (0.85, 0.98)	2.49	0.013	8	2927	3033	0.0%	0.967
homozygote (TT vs. CC)	overall	0.87 (0.76, 1.00)	1.89	0.058	10	3462	3547	0.0%	0.955
	hypertension	0.85 (0.70, 1.04)	1.59	0.111	4	1852	1787	0.0%	0.900
	CAD	0.99 (0.76, 1.30)	0.05	0.963	3	883	914	0.0%	0.609
	population-based	0.85 (0.70, 1.04)	1.99	0.047	8	2927	3033	0.0%	0.991
heterozygote (CT vs. CC)	overall/Asian	0.89 (0.80, 0.98)	2.26	0.024	10	3462	3547	0.0%	0.738
	hypertension	0.81 (0.70, 0.93)	2.95	0.003	4	1852	1787	0.0%	0.565
	CAD	0.98 (0.79, 1.21)	0.18	0.857	3	883	914	0.0%	0.977
	population-based	0.87 (0.78, 0.97)	2.46	0.014	8	2927	3033	0.0%	0.662
dominant (CT + TT vs. CC)	overall	0.88 (0.80, 0.97)	2.53	0.012	10	3462	3547	0.0%	0.866
	hypertension	0.82 (0.71, 0.93)	2.95	0.003	4	1852	1787	0.0%	0.650
	CAD	0.98 (0.80, 1.20)	0.16	0.871	3	883	914	0.0%	0.952
	population-based	0.86 (0.78, 0.96)	2.71	0.007	8	2927	3033	0.0%	0.821
recessive (TT vs. CC + CT)	overall/Asian	0.93 (0.82, 1.06)	1.03	0.303	10	3462	3547	0.0%	0.889
	hypertension	0.95 (0.80, 1.14)	0.52	0.601	4	1852	1787	0.0%	0.898
	CAD	1.01 (0.80, 1.28)	0.11	0.914	3	883	914	0.0%	0.483
	population-based	0.92 (0.80, 1.06)	1.14	0.256	8	2927	3033	0.0%	0.977

*OR* odds ratio, *CIs* confidence intervals, *CAD* coronary artery disease, *N* number of case-control studies

### The rs2108622 G/a polymorphism

**Fig. 3:**
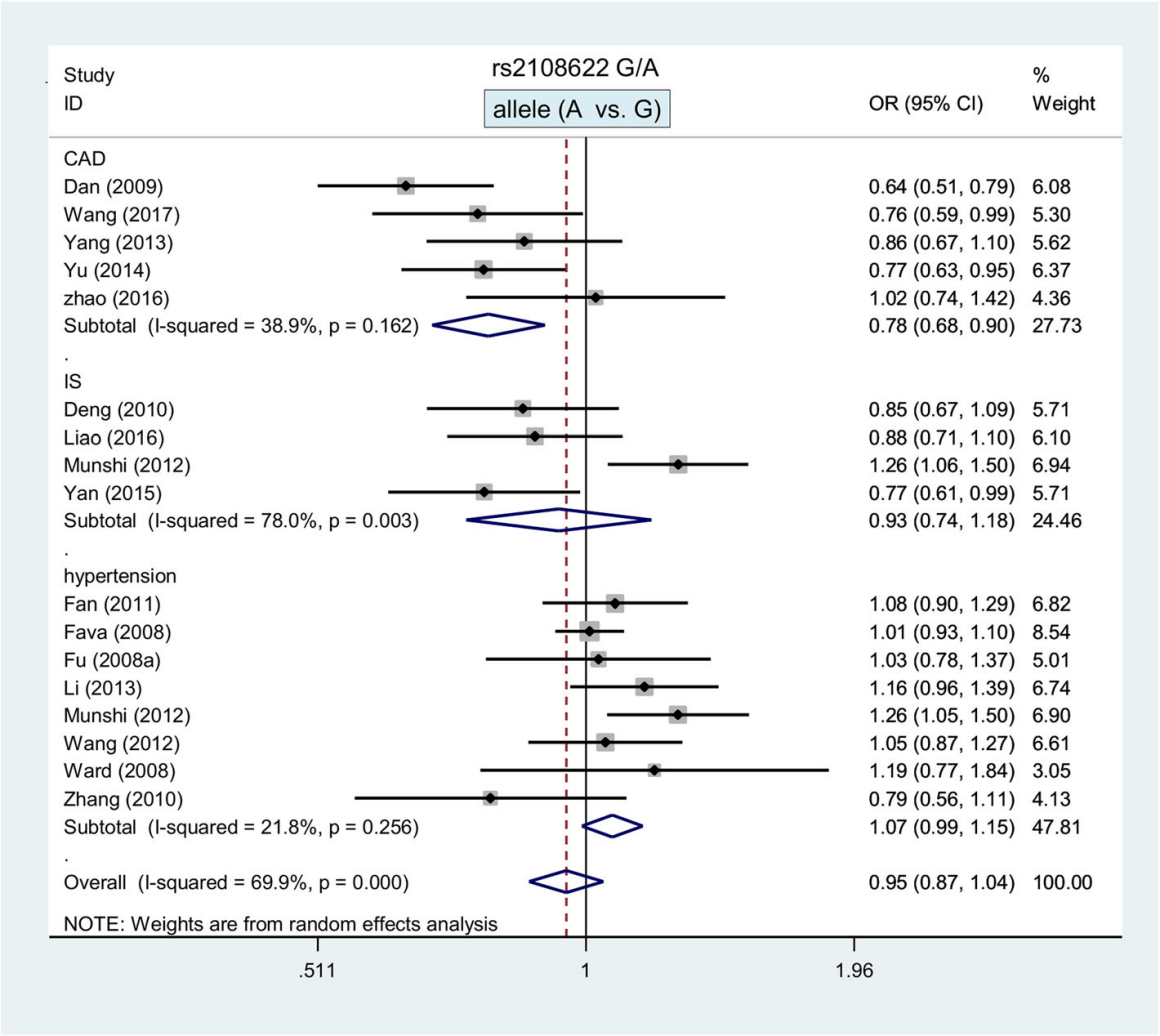
Subgroup analysis by disease type of the association between the *CYP4AF2* rs2108622 polymorphism and the risk of cardiovascular and cerebrovascular diseases under the allele model

Meta-analysis of the association between the *CYP4AF2* rs2108622 G/A polymorphism and the risk of cardiovascular and cerebrovascular diseases was subsequently performed. A total of 17 case-control studies were enrolled [8, 9, 17–20, 23, 24, 26–32, 34]. As shown in Table 2, a random-effects model was used in all meta-analysis (all *P* value of heterogeneity < 0.05). A decreased CAD risk was observed in the subgroup meta-analysis by disease type under all genetic models (Table 2, A vs. G, *P* value in association tests =0.001, OR = 0.78; AA vs. GG, *P* = 0.001, OR = 0.55; GA vs. GG, *P* = 0.002, OR = 0.80; GA + AA vs. GG, *P* = 0.005, OR = 0.77; AA vs. GG + GA, *P* = 0.002, OR = 0.60). However, no significant associations were observed for other factors (Table 2 and Additional file 1: Table S7). Figure 3 shows the forest plot of the subgroup analysis by disease type under the allele model, and Additional file 5: Figure S4, Additional file 6: Figure S5, Additional file 7: Figure S6 and Additional file 8: Figure S7 presents the data of homozygote, heterozygote, dominant and recessive models. Additional file 9: Figure S8 shows the forest plot of subgroup analysis by gender under the allele model. These data indicated that the *CYP4AF2* rs2108622 G/A polymorphism was associated with reduced susceptibility to CAD.

**Table 2:** Meta-analysis of *CYP4AF2* rs2108622 G/A polymorphism

Genetic models	Subgroup	Test of association			N	Sample size		Heterogeneity	
		OR (95% CIs)	z	*P* value		case	control	I^2^	*P* value
allele (A vs. G)	overall	0.95 (0.87, 1.04)	1.05	0.295	17	10,213	8033	69.9%	< 0.001
	Asian	0.95 (0.87, 1.04)	1.16	0.246	15	6352	5867	73.0%	< 0.001
	CAD	0.78 (0.68, 0.90)	3.40	0.001	5	1934	1609	38.9%	0.162
	IS	0.93 (0.74, 1.18)	0.58	0.564	4	1515	1545	78.0%	0.003
	hypertension	1.07 (0.99, 1.15)	1.69	0.090	8	6764	4879	21.8%	0.256
	population-based	0.92 (0.81, 1.05)	1.26	0.209	12	5203	4584	73.4%	< 0.001
homozygote (AA vs. GG)	overall	0.94 (0.76, 1.16)	0.58	0.561	17	10,213	8033	64.5%	< 0.001
	Asian	0.90 (0.71, 1.16)	0.81	0.418	15	6352	5867	67.9%	< 0.001
	CAD	0.55 (0.39, 0.77)	3.44	0.001	5	1934	1609	24.9%	0.255
	IS	0.90 (0.56, 1.44)	0.45	0.651	4	1515	1545	73.9%	0.009
	hypertension	0.90 (1.02, 1.37)	2.29	0.022	8	6764	4879	0.0%	0.525
	population-based	0.91 (0.70, 1.19)	0.70	0.487	12	5203	4584	64.3%	0.001
heterozygote (GA vs. GG)	overall	0.95 (0.89, 1.01)	1.69	0.092	17	10,213	8033	43.9%	0.027
	Asian	0.92 (0.85, 1.00)	2.06	0.039	15	6352	5867	48.3%	0.019
	CAD	0.80 (0.69, 0.92)	3.14	0.002	5	1934	1609	37.2%	0.173
	IS	0.94 (0.80, 1.09)	0.84	0.400	4	1515	1545	47.8%	0.125
	hypertension	1.00 (0.93, 1.09)	0.09	0.927	8	6764	4879	19.2%	0.278
	population-based	0.91 (0.83, 0.99)	2.15	0.032	12	5203	4584	57.8%	0.006
dominant (GA + AA vs. GG)	overall	0.93 (0.83, 1.03)	1.46	0.145	17	10,213	8033	59.3%	0.001
	Asian	0.91 (0.80, 1.03)	1.49	0.135	15	6352	5867	62.9%	0.001
	CAD	0.77 (0.64, 0.92)	2.83	0.005	5	1934	1609	43.3%	0.133
	IS	0.93 (0.71, 1.21)	0.57	0.572	4	1515	1545	69.4%	0.020
	hypertension	1.03 (0.95, 1.12)	0.77	0.442	8	6764	4879	6.0%	0.384
	population-based	0.89 (0.77, 1.03)	1.52	0.130	12	5203	4584	68.5%	< 0.001
recessive (AA vs. GG + GA)	overall	0.98 (0.82, 1.18)	0.22	0.827	17	10,213	8033	58.7%	0.001
	Asian	0.95 (0.77, 1.17)	0.49	0.625	15	6352	5867	62.5%	0.001
	CAD	0.60 (0.44, 0.83)	3.06	0.002	5	1934	1609	19.2%	0.293
	IS	0.93 (0.65, 1.33)	0.40	0.692	4	1515	1545	58.8%	0.064
	hypertension	1.22 (1.05, 1.43)	2.52	0.012	8	6764	4879	12.5%	0.333
	population-based	0.96 (0.77, 1.19)	0.39	0.694	12	5203	4584	50.5%	0.023

*OR* odds ratio, *CIs* confidence intervals, *CAD* coronary artery disease, *IS* ischemic stroke, *N* number of case-control studies

### Publication bias/sensitivity analysis

**Fig. 4:**
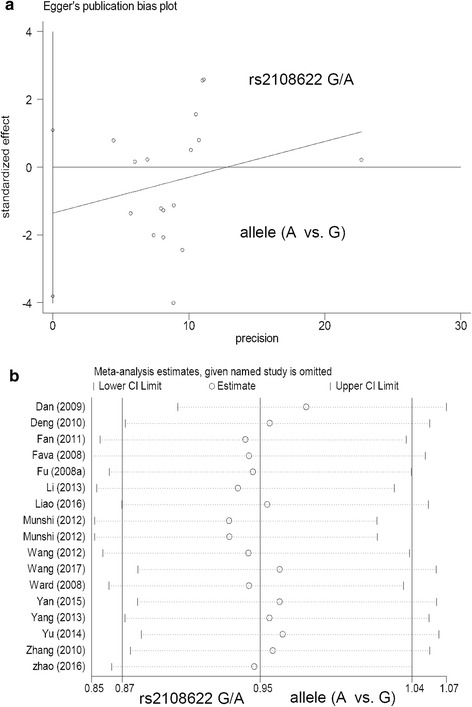
Begg’s test and sensitivity analysis for the *CYP4AF2* rs2108622 polymorphism under the allele model. **a** Funnel plot of Egger's test; **b** Sensitivity analysis

Table 3 shows the results of Begg’s/Egger’s tests under all genetic models (*P* value in Begg’s test > 0.05; *P* value in Egger’s test > 0.05), suggesting the absence of large publication bias. Fig. 4a presents the funnel plot of Egger's test under the allele model of the *CYP4AF2* rs2108622 G/A polymorphism. Furthermore, we observed the stability of the above data based on the sensitivity analysis (Fig. 4b for allele models of rs2108622, other models not shown).

**Table 3:** The assessment of publication bias

SNP	Comparison	Begg’s test		Egger’s test	
		z	*P* value	t	*P* value
rs1558139 C/T	allele (T vs. C)	0.89	0.371	−0.19	0.853
	homozygote (TT vs. CC)	1.07	0.283	−0.74	0.482
	heterozygote (CT vs. CC)	0.72	0.474	0.66	0.526
	dominant (CT + TT vs. CC)	0.89	0.371	0.63	0.549
	recessive (TT vs. CC + CT)	1.07	0.283	−1.33	0.221
rs2108622 G/A	allele (A vs. G)	1.69	0.091	−1.18	0.255
	homozygote (AA vs. GG)	1.28	0.202	−1.26	0.227
	heterozygote (GA vs. GG)	1.85	0.064	−1.55	0.143
	dominant (GA + AA vs. GG)	1.11	0.266	−1.18	0.257
	recessive (AA vs. GG + GA)	0.78	0.434	0.57	0.579

*SNP* single nucleotide polymorphism

## Discussion

*CYP4AF2* rs2108622 SNP was reportedly associated with an increased risk of hypertension cases in India [26] or Northern Han Chinese patients [23]. However, negative data were observed in another study in China [32]. In 2015, Luo et al. performed a meta-analysis of four studies [8, 19, 23, 32] and found that the *CYP4AF2* gene rs2108622 polymorphism was not associated with hypertension risk [13]. Here, eight case-control studies [8, 19, 20, 23, 26, 27, 29, 32] were included in our meta-analysis, and negative correlation between *CYP4AF2* rs2108622 and hypertension risk was observed. Nevertheless, we first investigated the role of *CYP4AF2* rs1558139 in the risk of hypertension. The findings suggested that the C/T genotype of *CYP4AF2* rs1558139 was correlated with a reduced risk of hypertension in the male patients of Asian populations. As a metabolite of arachidonic acid (AA), partly by CYP4F2 protein, 20-HETE induces the transcription of ACE (angiotensin-converting enzyme) gene through stimulating the NF-ΚB pathway and contributes to the pathophysiology of hypertension [10]. *CYP4AF2* rs1558139, located in an intron region, likely affects the enzyme activity of CYP4F2 protein during 20-HETE metabolism through altering the gene transcription level, RNA secondary structure or RNA splicing of the *CYP4AF2* gene, which is worth further investigation of the molecular mechanism. In addition, *CYP4F2* rs2108622 was tightly associated with systolic blood pressure as well as the excretion of 20-HETE [29]. Additional case-control studies are needed to further confirm the role of *CYP4F2* rs2108622 in hypertension risk.

Coronary heart disease results from the interplay between polygenic predisposition and complex environmental factors [35]. The *CYP4AF2* gene rs2108622 polymorphism was associated with the risk of coronary artery disease in a Han Chinese population [9]. However, a negative correlation between the *CYP4AF2* rs2108622 and coronary heart disease risk was reported in Mongolian cases in China [34]. In the present study, we first examined whether the *CYP4AF2* rs2108622 polymorphism is likely associated with the reduced susceptibility to coronary artery disease. Here, the meta-analysis provided evidence of the involvement of *CYP4AF2* rs2108622 in susceptibility to coronary heart disease. Given the reported correlation between *CYP4AF2* rs2108622 and 20-HETE [36], functional *CYP4AF2* rs2108622 may mediate the nonsynonymous mutation of a valine (V) located at residue 433 to a methionine (M), namely, V433 M, which then leads to the alteration of CYP4F2 protein function and 20-HETE synthesis, thereby influencing the occurrence of coronary heart disease.

The early evaluation of genetic risk factors is essential for the effective diagnosis and treatment of clinical ischemic stroke [3, 37]. We observed different conclusions for the association between *CYP4AF2* SNPs and ischemic stroke risk. For example, no detectable difference was reported by Liao, et al. for the genotype distribution of *CYP4AF2* rs2108622 between the control and cases of ischemic stroke [24]. However, Munshi, et al. reported *CYP4AF2* rs2108622 SNP as an important risk factor for ischemic stroke in an Indian population [26]. In 2015, Meng, et al. performed a meta-analysis of ischemic stroke and rs2108622 under the GA + AA vs. GG model [14], enrolling six studies [18, 20, 21, 26, 30, 38], and reported the data of OR> 1, *P* < 0.0001 [14]. In the present study, we removed two studies that did not contain the genotype data [20, 38] and one study on cerebral infarction [21], added one new study published in 2016 [24], and finally four studies [18, 24, 26, 30] were enrolled. Additionally, the GA + AA vs. GG model, and other genetic models, including A vs. G; AA vs. GG; GA vs. GG; AA vs. GG + GA, were utilized. We obtained a negative result for the genetic relationship between ischemic stroke risk and the *CYP4AF2* gene rs2108622 polymorphism. Different searches and analysis strategies might contribute to this finding.

We found that not all control data in these studies were population-based. After removing studies with hospital-based control data, the same positive correlation between the *CYP4AF2* rs1558139 C/T polymorphism and risk of cardiovascular and cerebrovascular diseases was obtained under allele, heterozygote and dominant genetic models. In addition, sensitivity analysis indicated the stability of the statistical results. Nevertheless, the small sample size of the included case-control studies, to a certain extent, limits the statistical power. Limited data were extracted for the *CYP4AF2* rs1558139 C/T polymorphism subgroup with the diseases of ischemic stroke or myocardial infarction.

Although no high degree of heterogeneity was observed in all meta-analyses of the *CYP4AF2* rs1558139 polymorphism, high heterogeneity was observed in most of the overall meta-analyses of *CYP4AF2* rs2108622. Apart from coronary heart disease and hypertension subgroup analyses, high heterogeneity still existed in other “Asian”, “ischemic stroke” and “population-based” subgroups. The complexity of disease feature might be the source of the large publication bias. In addition, unpublished data or articles in other languages may bias the selection of the studies analyzed.

Given the complicated etiologies in different types of cardiovascular and cerebrovascular diseases [4, 39], additional relative factors, such as obesity, age, comorbidities, smoking, and drinking should be assessed. We spared no effort to retrieve the relative articles using the general terms and the specific forms of heart, cardiovascular and cerebrovascular diseases (e.g., myocardial infarction, coronary artery disease, or hypertension, etc.). However, limited data of the above relative factors were obtained. Only four case-control studies [8, 19, 25, 27] were enrolled to perform the male/female subgroup analysis for the association between *CYP4AF2* rs1558139 and hypertension risk, while six studies [8, 19, 20, 23, 27, 32] were enrolled for *CYP4F2* rs2108622.

Adequate data on the genotype frequencies of both case and control studies are necessary to conduct SNP meta-analyses or subgroup analyses. Thus, only two SNPs (rs1558139, rs2108622) were studied in the present meta-analysis based on the available data. We did not examine the role of other variants within the *CYP4F2* gene, such as rs3093100, rs3093105, rs3093166, and rs3093135. The “G/G/G/T” haplotype of rs2108622-rs3093100-rs3093105-rs3093135 has been associated with an increased risk of coronary artery disease, however the “G/G/T/A” haplotype was associated with a reduced risk of coronary heart disease [9]. The potential distinct effect of different haplotypes merits further study. In addition, the combined effect of *CYP4F2* and other genes, such as *CYP4A11* (Cytochrome P450 Family 4 Subfamily A Member 11), should be analyzed upon the publication of sufficient data.

## Conclusions

Overall, the C/T genotype of the *CYP4AF2* rs1558139 polymorphism might serve as a protective factor for male patients with hypertension in Asian populations, and *CYP4AF2* rs2108622 may confer reduced genetic susceptibility to coronary heart disease.

## Additional files

Additional file 1: Table S1-S7. **Table S1.** Details of the search strategy based on four databases. **Table S2.** Basic information of the eligible studies in the meta-analysis. **Table S3.** Data of NOS assessment system. **Table S4.** Genotype frequencies of the eligible studies in the meta-analysis. **Table S5.** Genotype frequencies of the male/female group in some eligible studies. **Table S6.** Subgroup analysis by gender for the association between *CYP4AF2* rs1558139 and hypertension risk. **Table S7.** Subgroup analysis by gender for the association between *CYP4AF2* rs2108622 and hypertension risk. (DOCX 63 kb)

Additional file 2: Figure S1. Subgroup analysis by disease type of the association between the *CYP4AF2* rs1558139 polymorphism and the risk of cardiovascular and cerebrovascular diseases under the allele (T vs. C) model.. (TIFF 887 kb)

Additional file 3: Figure S2. Subgroup analysis by disease type of the association between the *CYP4AF2* rs1558139 polymorphism and the risk of cardiovascular and cerebrovascular diseases under the dominant (CT + TT vs. CC) model. (TIFF 967 kb)

Additional file 4: Figure S3. Subgroup analysis by gender of the association between the *CYP4AF2* rs1558139 polymorphism and the risk of cardiovascular and cerebrovascular diseases under the allele (T vs. C) model. (TIFF 1958 kb)

Additional file 5: Figure S4. Subgroup analysis by disease type of the association between the *CYP4AF2* rs2108622 polymorphism and the risk of cardiovascular and cerebrovascular diseases under the homozygote (AA vs. GG) model. (TIFF 1760 kb)

Additional file 6: Figure S5. Subgroup analysis by disease type of the association between the *CYP4AF2* rs2108622 polymorphism and the risk of cardiovascular and cerebrovascular diseases under the heterozygote (GA vs. GG) model. (TIFF 1717 kb)

Additional file 7: Figure S6. Subgroup analysis by disease type of the association between the *CYP4AF2* rs2108622 polymorphism and the risk of cardiovascular and cerebrovascular diseases under the dominant (GA + AA vs. GG) model. (TIFF 1689 kb)

Additional file 8: Figure S7. Subgroup analysis by disease type of the association between the *CYP4AF2* rs2108622 polymorphism and the risk of cardiovascular and cerebrovascular diseases under the recessive (AA vs. GG + GA) model. (TIFF 1792 kb)

Additional file 9: Figure S8. Subgroup analysis by gender of the association between the *CYP4AF2* rs2108622 polymorphism and the risk of cardiovascular and cerebrovascular diseases under the allele (A vs. G) model. (TIFF 1045 kb)

## Abbreviations

20-HETE: 20-hydroxy eicosane arachidonic acid
AA: Arachidonic acid
CAD: Coronary artery disease
CIs: Confidence intervals
CYP4A11: Cytochrome P450 Family 4 Subfamily A Member 11
CYP4F2: Cytochrome P450 Family 4 Subfamily F Member 2
IS: Ischemic stroke
MI: Myocardial infarction
OR: Odds ratio
PRISMA: Preferred Reporting Items for Systematic Reviews and Meta-Analyses
SNPs: Single nucleotide polymorphisms
