# Supplementary material for: Cytochrome P450 family 4 subfamily F member 2 (CYP4F2) rs1558139, rs2108622 polymorphisms and susceptibility to several cardiovascular and cerebrovascular diseases
Source: BMC Cardiovasc Disord. 2018 Feb 9;18:29. doi: 10.1186/s12872-018-0763-y (PMC5807755; doi:10.1186/s12872-018-0763-y)
Supplement: Supplementary file 1 — Table S1. Details of the search strategy based on four databases. Table S2. Basic information of the eligible studies in the meta-analysis. Table S3. Data of NOS assessment system. Table S4. Genotype frequencies of the eligible studies in the meta-analysis. Table S5. Genotype frequencies of the male/female group in some eligible studies. Table S6. Subgroup analysis by gender for the association between CYP4AF2 rs1558139 and hypertension risk. Table S7. Subgroup analysis by gender for the association between CYP4AF2 rs2108622 and hypertension risk. (DOCX 63 kb) [file 12872_2018_763_MOESM1_ESM.docx]

Table S1 Details of the search strategy based on four databases

| Database name | Searching terms | Number |
| --- | --- | --- |
| [1]PubMed | @1= (((((((((((((((((((cerebralvascular diseases) OR cerebralvascular disorders) OR cardiovascular diseases) OR cardiovascular disorders) OR cardio-cerebral vascular diseases) OR cardio-cerebral vascular disorders) OR myocardial infarction) OR hypertension) OR coronary artery disease) OR ischemic stroke) OR heart failure) OR cardiac arrhythmia) OR rheumatic heart disease) OR hyperlipidemia) OR cerebral infarction) OR cerebral atherosclerosis) OR cerebral atherosclerosis) OR cerebral arteritis) OR cerebral aneurysm) OR intracranial vascular malformations | 2,503,510 |
|  | @2=(((((((((((((((CYP4F2) OR Cytochrome P450 Family 4 Subfamily F Member 2) OR Cytochrome P450, Family 4, Subfamily F, Polypeptide 2) OR Cytochrome P450, Subfamily IVF, Polypeptide 2) OR 20-Hydroxyeicosatetraenoic Acid Synthase) OR Leukotriene-B(4) Omega-Hydroxylase 1) OR Leukotriene-B(4) 20-Monooxygenase 1) OR Arachidonic Acid Omega-Hydroxylase) OR Cytochrome P450-LTB-Omega) OR Cytochrome P450 4F2) OR 20-HETE Synthase) OR CYPIVF2) OR Phylloquinone Omega-Hydroxylase CYP4F2) OR EC 1.14.13.194) OR EC 1.14.13.30) OR CPF2 | 432 |
|  | @3=@1 and @2 | 128 |
| [2] WOS | @1=TOPIC: (cerebralvascular diseases) OR TOPIC: (cardiovascular diseases) OR TOPIC: (cardiovascular disorders) OR TOPIC: (cardio-cerebral vascular diseases) OR TOPIC: (cardio-cerebral vascular disorders) OR TOPIC: (myocardial infarction) OR TOPIC: (hypertension) OR TOPIC: (coronary artery disease) OR TOPIC: (ischemic stroke) OR TOPIC: (heart failure) OR TOPIC: (cardiac arrhythmia) OR TOPIC: (rheumatic heart disease) OR TOPIC: (hyperlipidemia) OR TOPIC: (cerebral infarction) OR TOPIC: (cerebral atherosclerosis) OR TOPIC: (cerebral atherosclerosis) OR TOPIC: (cerebral arteritis) OR TOPIC: (cerebral aneurysm) OR TOPIC: (intracranial vascular malformations)  Timespan=1980-2018  Search language=Auto | 1,773,989 |
|  | @2=TOPIC: (CYP4F2) OR TOPIC: (Cytochrome P450 Family 4 Subfamily F Member 2) OR TOPIC: (Cytochrome P450, Family 4, Subfamily F, Polypeptide 2) OR TOPIC: (Cytochrome P450, Subfamily IVF, Polypeptide 2) OR TOPIC: (20-Hydroxyeicosatetraenoic Acid Synthase) OR TOPIC: (Leukotriene-B(4) Omega-Hydroxylase 1) OR TOPIC: (Leukotriene-B(4) 20-Monooxygenase 1) OR TOPIC: (Arachidonic Acid Omega-Hydroxylase) OR TOPIC: (Cytochrome P450-LTB-Omega) OR TOPIC: (Cytochrome P450 4F2) OR TOPIC: (20-HETE Synthase) OR TOPIC: (CYPIVF2) OR TOPIC: (Phylloquinone Omega-Hydroxylase CYP4F2) OR TOPIC: (EC 1.14.13.194) OR TOPIC: (EC 1.14.13.30) OR TOPIC: (CPF2)  Timespan=1980-2018  Search language=Auto | 909 |
|  | @3=@1 and @2  Timespan=1980-2018  Search language=Auto | 369 |
| [3]CNKI | TOPIC=CYP4F2 and TOPIC = Polymorphism | 36 |
| [4] WANFANG DATA | CYP4F2 * Polymorphism * Date:-2018 | 64 |

***WOS* Web of Science, *CNK*I China National Knowledge Infrastructure**

Table S2 Basic information of the eligible studies in the meta-analysis.

| First author | Year | Ethnicity | assay | Disease | SNP | case | | | control | | |
| --- | --- | --- | --- | --- | --- | --- | --- | --- | --- | --- | --- |
|  |  |  |  |  |  | number | M (%) | Age* | number | M (%) | Age* |
| Dan | 2009 | Asian | PCR-RFLP | CAD | rs2108622 G/A | 420 | 71.4% | 61.8 | 412 | 62.1% | 60.6 |
| Deng | 2010 | Asian | PCR-RFLP | IS | rs2108622 G/A | 302 | 63.9% | NA | 350 | 60.6% | NA |
|  |  |  |  |  | rs1558139 C/T | 302 | 63.9% | NA | 350 | 60.6% | NA |
| Fan | 2011 | Asian | Taqman PCR | hypertension | rs1558139 C/T | 677 | 45.2% | NA | 654 | 44.6% | NA |
|  |  |  |  |  | rs2108622 G/A | 680 | 45.1% | NA | 653 | 44.7% | NA |
| Fava | 2008 | Caucasian | end-point fluorescent measurements | hypertension | rs2108622 G/A | 3700 | 45.7% | NA | 2092 | 35.9% | NA |
| Fu | 2008a | Asian | Taqman PCR | hypertension | rs1558139 C/T | 249 | 66.3% | 51.1 | 238 | 65.1% | 51.3 |
|  |  |  |  |  | rs2108622 G/A | 249 | 66.3% | 51.1 | 238 | 65.1% | 51.3 |
| Fu | 2008b | Asian | Taqman PCR | CI | rs1558139 C/T | 175 | 60.0% | 66.2 | 246 | 50.0% | 77.5 |
| Huang | 2009 | Asian | Taqman PCR | MI | rs1558139 C/T | 250 | 78.8% | 61.8 | 250 | 50.0% | 62.5 |
| Li | 2013 | Asian | Taqman PCR | hypertension | rs2108622 G/A | 760 | 63.3% | 50.1 | 470 | 59.8% | 50.4 |
| Liao | 2016 | Asian | MALDI-TOF | IS | rs2108622 G/A | 396 | 59.3% | 68.8 | 378 | 58.7% | 65.0 |
| Liu | 2012 | Asian | HRM analysis-gene sequencing | hypertension | rs1558139 C/T | 328 | 57.3% | NA | 297 | 49.2% | NA |
|  |  |  |  | CAD | rs1558139 C/T | 364 | 71.2% | NA | 429 | 47.1% | NA |
| Munshi | 2012 | Asian | PCR-RFLP | IS | rs2108622 G/A | 507 | 71.6% | 49.3 | 487 | 73.1% | 49.0 |
|  |  |  |  | hypertension | rs2108622 G/A | 427 | NA | NA | 567 | NA | NA |
| Wang | 2012 | Asian | Taqman PCR | hypertension | rs1558139 C/T | 598 | 47.3% | NA | 598 | 45.0% | NA |
|  |  |  |  |  | rs2108622 G/A | 598 | 47.3% | NA | 598 | 45.0% | NA |
| Wang | 2017 | Asian | Sequenom Mass ARRAY | CAD | rs2108622 G/A | 514 | 66.0% | 58.9 | 198 | 72.2% | 56.2 |
| Ward | 2008 | Caucasian | gene sequencing | hypertension | rs2108622 G/A | 161 | 60.2% | 58.2 | 74 | 43.2% | 55.6 |
| Yan | 2015 | Asian | PCR-RFLP | IS | rs2108622 G/A | 310 | 61.3% | 57.8 | 330 | 64.0% | 58.5 |
| Yang | 2013 | Asian | Taqman PCR | CAD | rs2108622 G/A | 326 | NA | 63.1 | 338 | NA | 63.2 |
| Yu | 2014 | Asian | PCR-RFLP | CAD | rs2108622 G/A | 440 | NA | 58.6 | 440 | NA | 58.5 |
| Zhang | 2010 | Asian | PCR-RFLP | hypertension | rs2108622 G/A | 189 | 46.0% | 62.1 | 187 | 54.0% | 63.3 |
| zhao | 2017 | Asian | LDR | CAD | rs1558139 C/T | 285 | 65.6% | 63.1 | 264 | 45.1% | 60.6 |
| zhao | 2016 | Asian | LDR | CAD | rs2108622 G/A | 234 | 68.0% | 62.1 | 221 | 41.2% | 59.6 |
|  |  |  |  |  | rs1558139 C/T | 234 | 68.0% | 62.1 | 221 | 41.2% | 59.6 |

***PCR* Polymerase Chain Reaction, *RFLP* restriction fragment length polymorphism, *HRM* high-resolution melting, *LDR* ligase detection reaction, *CAD* coronary artery disease, *IS* ischemic stroke, *CI* cerebral infarction, *MI* myocardial infarction, *SNP* single nucleotide polymorphism, *M* male,**

***** mean age (year), *NA* not available**

Table S3 Data of NOS assessment system

| First author | Year | Score | Case | | Control | | Comparability | | Exposure | | |
| --- | --- | --- | --- | --- | --- | --- | --- | --- | --- | --- | --- |
|  |  |  | Adequate  definition | Consecutive or obvious representativeness | Community controls | No history of disease | Most important factor | Any additional factors | Secure record | Same method | Non-response rate |
| Dan | 2009 | 8 | 1 | 1 | 1 | 1 | 1 | 0 | 1 | 1 | 1 |
| Deng | 2010 | 9 | 1 | 1 | 1 | 1 | 1 | 1 | 1 | 1 | 1 |
| Fan | 2011 | 6 | 0 | 1 | 1 | 1 | 1 | 0 | 1 | 1 | 0 |
| Fava | 2008 | 6 | 1 | 1 | 0 | 0 | 1 | 0 | 1 | 1 | 1 |
| Fu | 2008a | 8 | 1 | 1 | 1 | 1 | 1 | 0 | 1 | 1 | 1 |
| Fu | 2008b | 8 | 1 | 1 | 1 | 1 | 1 | 0 | 1 | 1 | 1 |
| Huang | 2009 | 6 | 1 | 1 | 0 | 0 | 1 | 0 | 1 | 1 | 1 |
| Li | 2013 | 9 | 1 | 1 | 1 | 1 | 1 | 1 | 1 | 1 | 1 |
| Liao | 2016 | 6 | 1 | 1 | 0 | 0 | 1 | 0 | 1 | 1 | 1 |
| Liu | 2012 | 8 | 1 | 1 | 1 | 1 | 1 | 0 | 1 | 1 | 1 |
| Munshi | 2012 | 9 | 1 | 1 | 1 | 1 | 1 | 1 | 1 | 1 | 1 |
| Wang | 2012 | 7 | 0 | 1 | 1 | 1 | 1 | 0 | 1 | 1 | 1 |
| Wang | 2017 | 6 | 0 | 0 | 1 | 1 | 1 | 0 | 1 | 1 | 1 |
| Ward | 2008 | 6 | 1 | 1 | 0 | 0 | 1 | 0 | 1 | 1 | 1 |
| Yan | 2015 | 9 | 1 | 1 | 1 | 1 | 1 | 1 | 1 | 1 | 1 |
| Yang | 2013 | 7 | 1 | 1 | 0 | 0 | 1 | 1 | 1 | 1 | 1 |
| Yu | 2014 | 9 | 1 | 1 | 1 | 1 | 1 | 1 | 1 | 1 | 1 |
| Zhang | 2010 | 8 | 1 | 1 | 1 | 1 | 1 | 0 | 1 | 1 | 1 |
| zhao | 2017 | 6 | 1 | 1 | 0 | 0 | 1 | 0 | 1 | 1 | 1 |
| zhao | 2016 | 8 | 1 | 1 | 1 | 1 | 1 | 0 | 1 | 1 | 1 |

***NOS* Newcastle-Ottawa Scale**

Table S4 Genotype frequencies of the eligible studies in the meta-analysis.

| First author | Year | SNP | Disease | case | | | control | | | HWE | | Source of control |
| --- | --- | --- | --- | --- | --- | --- | --- | --- | --- | --- | --- | --- |
|  |  |  |  | XX | Xx | xx | XX | Xx | xx | X^2^ | *P* value |  |
| Dan | 2009 | rs2108622 G/A | CAD | 252 | 152 | 16 | 193 | 186 | 33 | 1.65 | 0.20 | population-based |
| Deng | 2010 | rs2108622 G/A | IS | 161 | 117 | 24 | 171 | 144 | 35 | 0.33 | 0.56 | population-based |
|  |  | rs1558139 G/A | IS | 119 | 134 | 49 | 129 | 158 | 63 | 1.43 | 0.23 | population-based |
| Fan | 2011 | rs1558139 C/T | hypertension | 247 | 321 | 109 | 220 | 325 | 109 | 0.36 | 0.55 | population-based |
|  |  | rs2108622 G/A | hypertension | 410 | 226 | 44 | 403 | 215 | 35 | 0.80 | 0.37 | population-based |
| Fava | 2008 | rs2108622 G/A | hypertension | 2014 | 1435 | 251 | 1142 | 812 | 138 | 0.15 | 0.69 | population/ hospital -based |
| Fu | 2008a | rs1558139 C/T | hypertension | 125 | 93 | 31 | 97 | 113 | 28 | 0.32 | 0.57 | population-based |
|  |  | rs2108622 G/A | hypertension | 143 | 74 | 32 | 128 | 91 | 19 | 0.25 | 0.62 | population-based |
| Fu | 2008b | rs1558139 C/T | CI | 80 | 77 | 18 | 110 | 102 | 34 | 1.70 | 0.19 | population-based |
| Huang | 2009 | rs1558139 C/T | MI | 115 | 109 | 26 | 113 | 102 | 35 | 2.30 | 0.13 | hospital-based |
| Li | 2013 | rs2108622 G/A | hypertension | 403 | 296 | 61 | 273 | 162 | 35 | 2.50 | 0.11 | population-based |
| Liao | 2016 | rs2108622 G/A | IS | 209 | 155 | 32 | 186 | 155 | 37 | 0.32 | 0.57 | hospital-based |
| Liu | 2012 | rs1558139 C/T | hypertension | 137 | 150 | 41 | 105 | 148 | 44 | 0.49 | 0.48 | population-based |
|  |  | rs1558139 C/T | CAD | 140 | 177 | 47 | 160 | 206 | 63 | 0.06 | 0.80 | population-based |
| Munshi | 2012 | rs2108622 G/A | IS | 114 | 257 | 136 | 138 | 246 | 103 | 0.12 | 0.73 | population-based |
|  |  | rs2108622 G/A | hypertension | 98 | 203 | 126 | 147 | 301 | 119 | 2.35 | 0.13 | population/ hospital -based |
| Wang | 2012 | rs1558139 C/T | hypertension | 225 | 278 | 95 | 201 | 300 | 97 | 0.72 | 0.40 | population-based |
|  |  | rs2108622 G/A | hypertension | 364 | 197 | 37 | 370 | 195 | 33 | 1.17 | 0.28 | population-based |
| Wang | 2017 | rs2108622 G/A | CAD | 306 | 183 | 25 | 104 | 78 | 16 | 0.07 | 0.80 | population-based |
| Ward | 2008 | rs2108622 G/A | hypertension | 82 | 62 | 17 | 39 | 31 | 4 | 0.47 | 0.50 | NA |
| Yan | 2015 | rs2108622 G/A | IS | 167 | 118 | 25 | 152 | 142 | 36 | 0.11 | 0.74 | population-based |
| Yang | 2013 | rs2108622 G/A | CAD | 177 | 138 | 11 | 178 | 134 | 26 | 0.01 | 0.91 | hospital-based |
| Yu | 2014 | rs2108622 G/A | CAD | 238 | 168 | 34 | 203 | 191 | 46 | 0.01 | 0.91 | population-based |
| Zhang | 2010 | rs2108622 G/A | hypertension | 122 | 55 | 12 | 105 | 70 | 12 | 0.01 | 0.94 | population-based |
| zhao | 2017 | rs1558139 C/T | CAD | 81 | 137 | 67 | 76 | 135 | 53 | 0.25 | 0.62 | hospital-based |
| zhao | 2016 | rs2108622 G/A | CAD | 148 | 77 | 9 | 139 | 76 | 6 | 1.36 | 0.24 | population-based |
|  |  | rs1558139 C/T | CAD | 61 | 108 | 65 | 58 | 101 | 62 | 1.62 | 0.20 | population-based |

***SNP* single nucleotide polymorphism, *CAD* coronary artery disease, *IS* ischemic stroke, *CI* cerebral infarction, *MI* myocardial infarction, *X* major allele, *x* minor allele, *NA* not available, *HWE* Hardy-Weinberg Equilibrium**

Table S5 Genotype frequencies of the male/female group in some eligible studies.

| First | Year | SNP | Disease-gender | case | | | control | | | HWE | | Source of control |
| --- | --- | --- | --- | --- | --- | --- | --- | --- | --- | --- | --- | --- |
| author |  |  |  | XX | Xx | xx | XX | Xx | xx | X^2^ | *P* value |  |
| Deng | 2010 | rs2108622 G/A | IS-male | 112 | 71 | 10 | 102 | 87 | 23 | 0.47 | 0.49 | population-based |
|  |  |  | IS-female | 49 | 46 | 14 | 69 | 57 | 12 | 0.00 | 0.96 | population-based |
|  |  | rs1558139 C/T | IS-male | 80 | 82 | 31 | 79 | 94 | 39 | 1.37 | 0.24 | population-based |
|  |  |  | IS-female | 39 | 52 | 18 | 50 | 64 | 24 | 0.20 | 0.65 | population-based |
| Fan | 2011 | rs1558139 C/T | hypertension-male | 116 | 144 | 46 | 90 | 154 | 48 | 1.73 | 0.19 | population-based |
|  |  |  | hypertension-female | 131 | 177 | 63 | 130 | 171 | 61 | 0.14 | 0.71 | population-based |
|  |  | rs2108622 G/A | hypertension-male | 195 | 96 | 16 | 172 | 102 | 18 | 0.30 | 0.58 | population-based |
|  |  |  | hypertension-female | 215 | 130 | 28 | 231 | 113 | 17 | 0.44 | 0.51 | population-based |
| Fava | 2008 | rs2108622 G/A | hypertension-male | 879 | 683 | 128 | 422 | 283 | 47 | 0.00 | 0.96 | population/ hospital -based |
|  |  |  | hypertension-female | 1135 | 752 | 123 | 720 | 529 | 91 | 0.22 | 0.64 | population/ hospital -based |
| Fu | 2008a | rs1558139 C/T | hypertension-male | 92 | 54 | 19 | 62 | 74 | 19 | 0.18 | 0.67 | population-based |
|  |  |  | hypertension-female | 33 | 39 | 12 | 35 | 39 | 9 | 0.15 | 0.70 | population-based |
|  |  | rs2108622 G/A | hypertension-male | 95 | 50 | 20 | 86 | 58 | 11 | 0.08 | 0.78 | population-based |
|  |  |  | hypertension-female | 48 | 24 | 12 | 42 | 33 | 8 | 0.16 | 0.69 | population-based |
| Fu | 2008b | rs1558139 C/T | CI-male | 52 | 43 | 10 | 47 | 58 | 18 | 0.00 | 0.99 | population-based |
|  |  |  | CI-female | 28 | 34 | 8 | 63 | 44 | 16 | 3.24 | 0.07 | population-based |
| Huang | 2009 | rs1558139 C/T | MI-male | 96 | 81 | 20 | 49 | 57 | 19 | 0.13 | 0.72 | hospital-based |
|  |  |  | MI-female | 19 | 28 | 6 | 64 | 45 | 16 | 3.02 | 0.08 | hospital-based |
| Li | 2013 | rs2108622 G/A | hypertension-male | 255 | 187 | 39 | 166 | 97 | 18 | 0.56 | 0.45 | population-based |
|  |  |  | hypertension-female | 148 | 109 | 22 | 107 | 65 | 17 | 2.31 | 0.13 | population-based |
| Liu | 2012 | rs1558139 C/T | hypertension-male | 84 | 86 | 18 | 51 | 73 | 22 | 0.25 | 0.62 | population-based |
|  |  |  | hypertension-female | 53 | 64 | 23 | 54 | 75 | 22 | 0.24 | 0.62 | population-based |
|  |  |  | CAD-male | 102 | 128 | 29 | 76 | 98 | 28 | 0.16 | 0.69 | population-based |
|  |  |  | CAD-female | 38 | 50 | 17 | 84 | 108 | 35 | 0.00 | 0.98 | population-based |
| Wang | 2012 | rs1558139 C/T | hypertension-male | 112 | 130 | 41 | 86 | 140 | 43 | 1.25 | 0.26 | population-based |
|  |  |  | hypertension-female | 113 | 148 | 54 | 115 | 160 | 54 | 0.02 | 0.90 | population-based |
|  |  | rs2108622 G/A | hypertension-male | 184 | 85 | 14 | 158 | 96 | 16 | 0.08 | 0.78 | population-based |
|  |  |  | hypertension-female | 180 | 112 | 23 | 212 | 100 | 17 | 1.30 | 0.25 | population-based |
| Zhang | 2010 | rs2108622 G/A | hypertension-male | 58 | 26 | 3 | 50 | 44 | 7 | 0.42 | 0.52 | population-based |
|  |  |  | hypertension-female | 64 | 29 | 9 | 55 | 26 | 5 | 0.64 | 0.42 | population-based |

***SNP* single nucleotide polymorphism, *IS* ischemic stroke, *CAD* coronary artery disease, *CI* cerebral infarction, *MI* myocardial infarction, *X* major allele, *x* minor allele, *HWE* Hardy-Weinberg Equilibrium**

Table S6 Subgroup analysis based on gender factor for the association between *CYP4AF2* rs1558139 and hypertension risk.

| Genetic models | Subgroup | Test of association | | | N | Sample size | | Heterogeneity | |
| --- | --- | --- | --- | --- | --- | --- | --- | --- | --- |
|  |  | OR (95% CIs) | z | *P* value |  | case | control | I^2^ | *P* value |
| allele (T vs. C) | male | 0.79 (0.69, 0.90) | 3.51 | <0.001 | 4 | 942 | 862 | 0.0% | 0.694 |
|  | female | 1.02 (0.89, 1.16) | 0.24 | 0.813 | 4 | 910 | 925 | 0.0% | 0.954 |
| homozygote (TT vs. CC) | male | 0.68 (0.51, 0.91) | 2.60 | 0.009 | 4 | 942 | 862 | 0.0% | 0.813 |
|  | female | 1.05 (0.80, 1.39) | 0.38 | 0.704 | 4 | 910 | 925 | 0.0% | 0.944 |
| heterozygote (CT vs. CC) | male | 0.67 (0.55, 0.82) | 3.86 | <0.001 | 4 | 942 | 862 | 0.0% | 0.573 |
|  | female | 0.97 (0.80, 1.19) | 0.26 | 0.793 | 4 | 910 | 925 | 0.0% | 0.939 |
| dominant (CT+TT vs. CC) | male | 0.67 (0.55, 0.81) | 4.05 | <0.001 | 4 | 942 | 862 | 0.0% | 0.688 |
|  | female | 0.99 (0.82, 1.20) | 0.07 | 0.946 | 4 | 910 | 925 | 0.0% | 0.947 |
| recessive (TT vs. CC+CT) | male | 0.84 (0.65, 1.10) | 1.25 | 0.210 | 4 | 942 | 862 | 0.0% | 0.739 |
|  | female | 1.07 (0.83, 1.37) | 0.53 | 0.595 | 4 | 910 | 925 | 0.0% | 0.936 |

***OR* odds ratio, *CIs* confidence intervals, *N* number of case-control studies**

Table S7 Subgroup analysis based on gender factor for the association between *CYP4AF2* rs2108622 and hypertension risk.

| Genetic models | Subgroup | Test of association | | | N | Sample size | | Heterogeneity | |
| --- | --- | --- | --- | --- | --- | --- | --- | --- | --- |
|  |  | OR (95% CIs) | z | *P* |  | case | control | I^2^ | *P* value |
| allele (A vs. G) | male | 0.96 (0.79, 1.16) | 0.40 | 0.688 | 6 | 3,013 | 1,851 | 67.2% | 0.009 |
|  | female | 1.09 (0.93, 1.29) | 1.07 | 0.285 | 6 | 3,163 | 2,388 | 55.4% | 0.047 |
| homozygote (AA vs. GG) | male | 1.11 (0.82, 1.51) | 0.67 | 0.506 | 6 | 3,013 | 1,851 | 24.7% | 0.249 |
|  | female | 1.15 (0.86, 1.55) | 0.94 | 0.347 | 6 | 3,163 | 2,388 | 26.9% | 0.233 |
| heterozygote (GA vs. GG) | male | 1.00 (0.89, 1.14) | 0.07 | 0.946 | 6 | 3,013 | 1,851 | 63.1% | 0.019 |
|  | female | 1.00 (0.89, 1.12) | 0.03 | 0.976 | 6 | 3,163 | 2,388 | 44.5% | 0.109 |
| dominant (GA+AA vs. GG) | male | 0.92 (0.73, 1.17) | 0.66 | 0.512 | 6 | 3,013 | 1,851 | 67.8% | 0.008 |
|  | female | 1.08 (0.89, 1.32) | 0.81 | 0.417 | 6 | 3,163 | 2,388 | 52.0% | 0.064 |
| recessive (AA vs. GG+GA | male | 1.15 (0.90, 1.45) | 1.13 | 0.258 | 6 | 3,013 | 1,851 | 0.0% | 0.451 |
|  | female | 1.06 (0.86, 1.32) | 0.57 | 0.569 | 6 | 3,163 | 2,388 | 11.6% | 0.341 |

***OR* odds ratio, *CIs* confidence intervals, *N* number of case-control studies**
