# Supplementary figures and images for: Cytochrome P450 family 4 subfamily F member 2 (CYP4F2) rs1558139, rs2108622 polymorphisms and susceptibility to several cardiovascular and cerebrovascular diseases
Source: BMC Cardiovasc Disord. 2018 Feb 9;18:29. doi: 10.1186/s12872-018-0763-y (PMC5807755; doi:10.1186/s12872-018-0763-y)

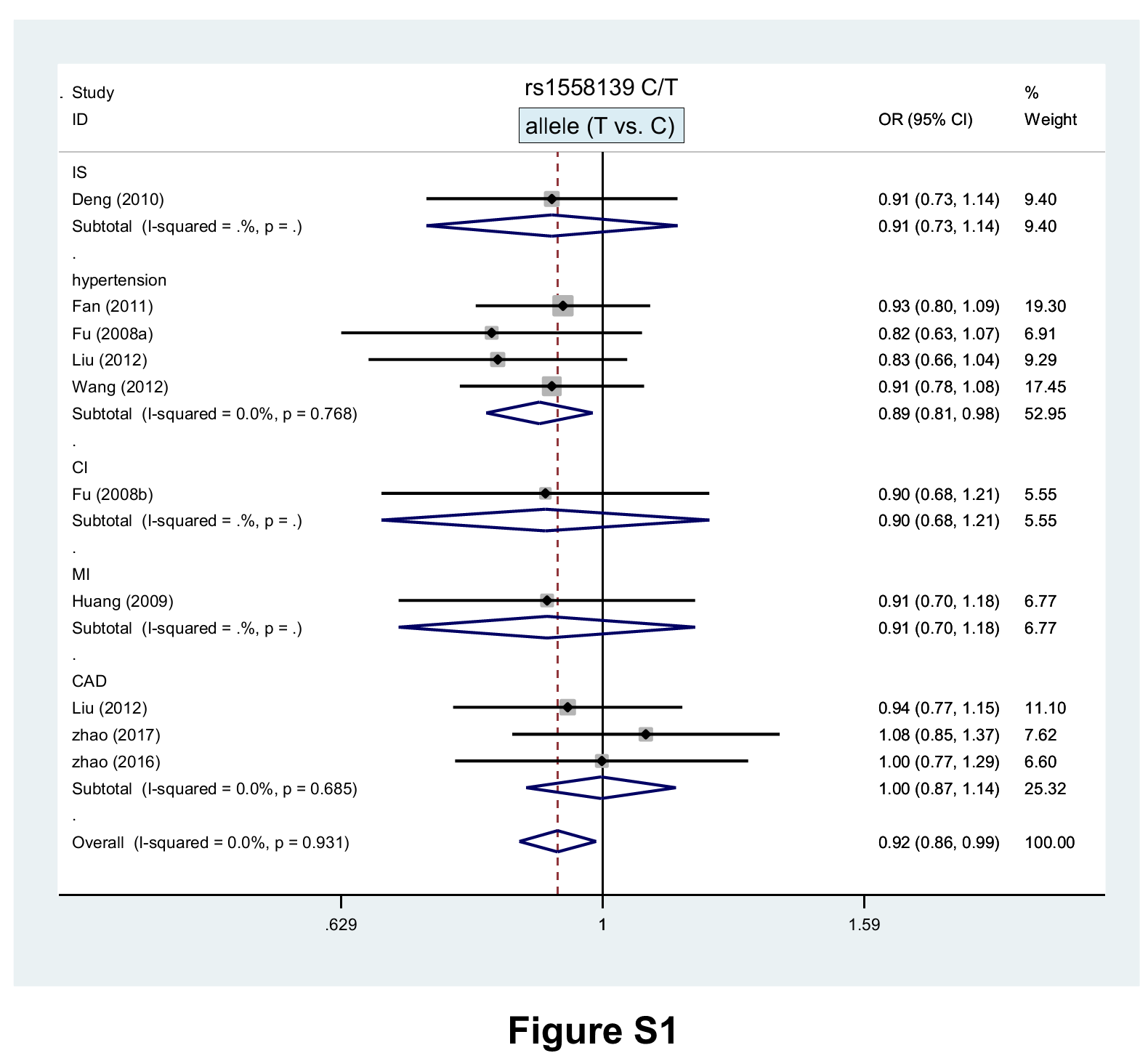

Supplement: Supplementary file 2 — Subgroup analysis by disease type of the association between the CYP4AF2 rs1558139 polymorphism and the risk of cardiovascular and cerebrovascular diseases under the allele (T vs. C) model.. (TIFF 887 kb) [file 12872_2018_763_MOESM2_ESM.tif]

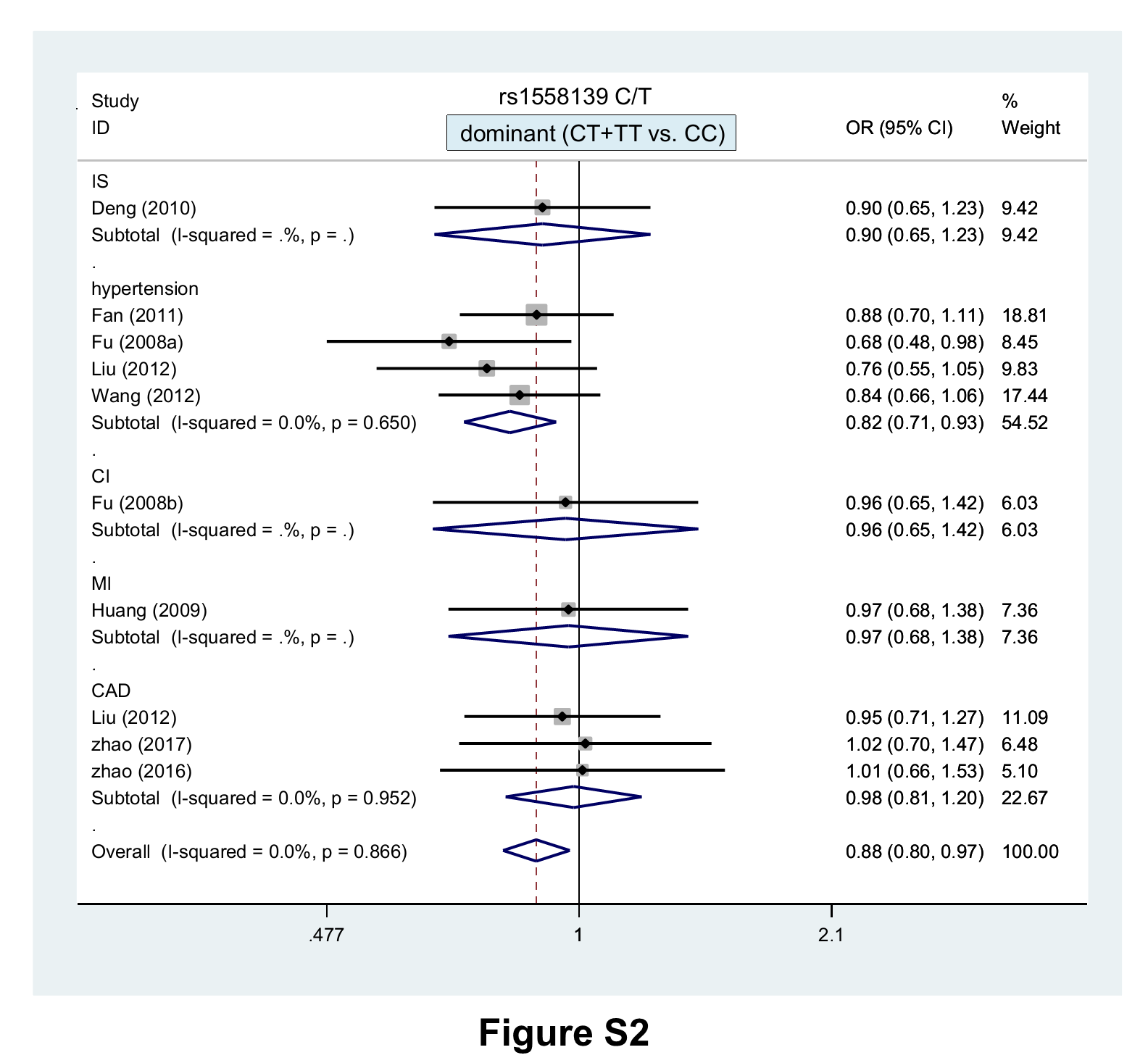

Supplement: Supplementary file 3 — Subgroup analysis by disease type of the association between the CYP4AF2 rs1558139 polymorphism and the risk of cardiovascular and cerebrovascular diseases under the dominant (CT + TT vs. CC) model. (TIFF 967 kb) [file 12872_2018_763_MOESM3_ESM.tif]

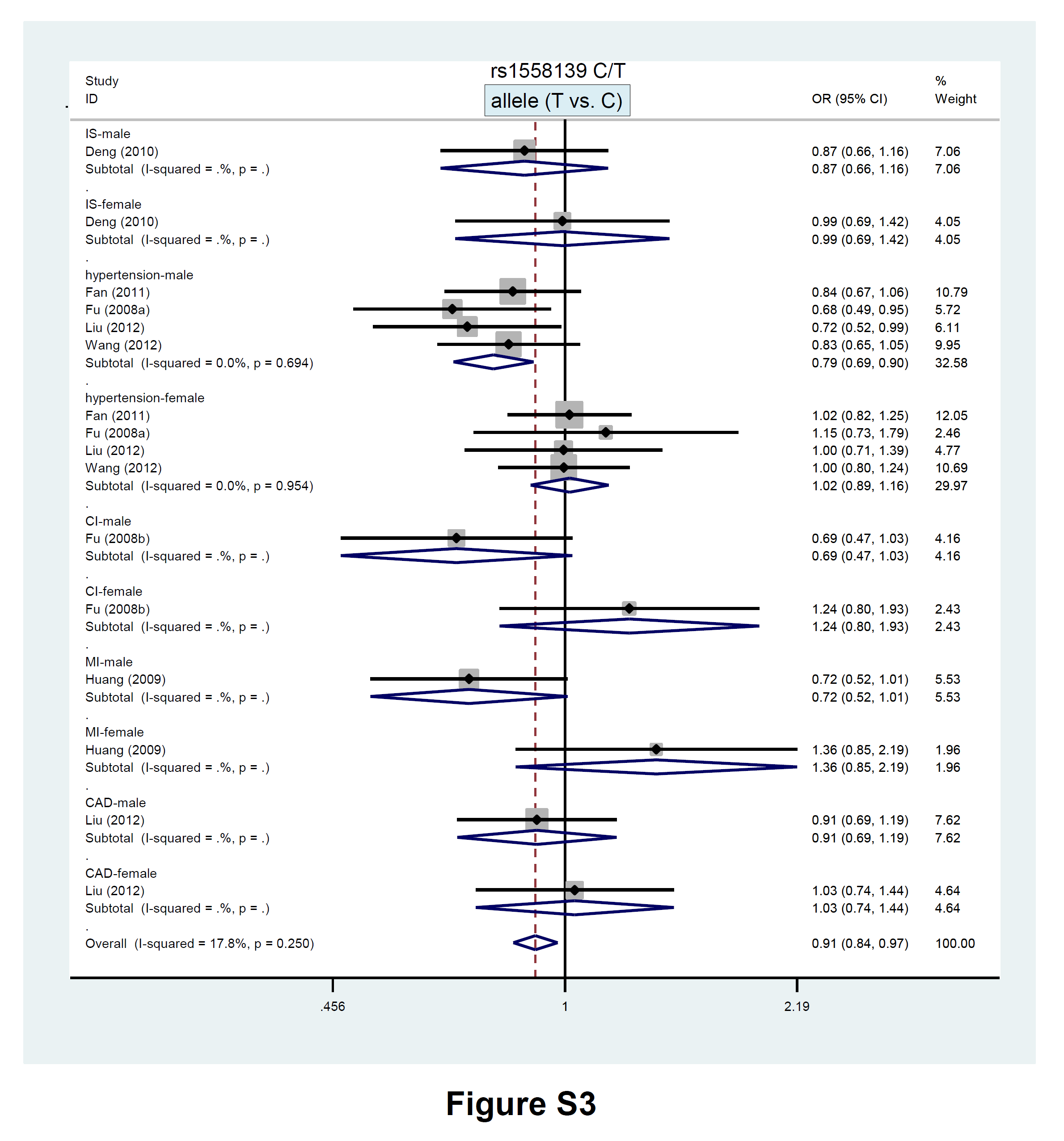

Supplement: Supplementary file 4 — Subgroup analysis by gender of the association between the CYP4AF2 rs1558139 polymorphism and the risk of cardiovascular and cerebrovascular diseases under the allele (T vs. C) model. (TIFF 1958 kb) [file 12872_2018_763_MOESM4_ESM.tif]

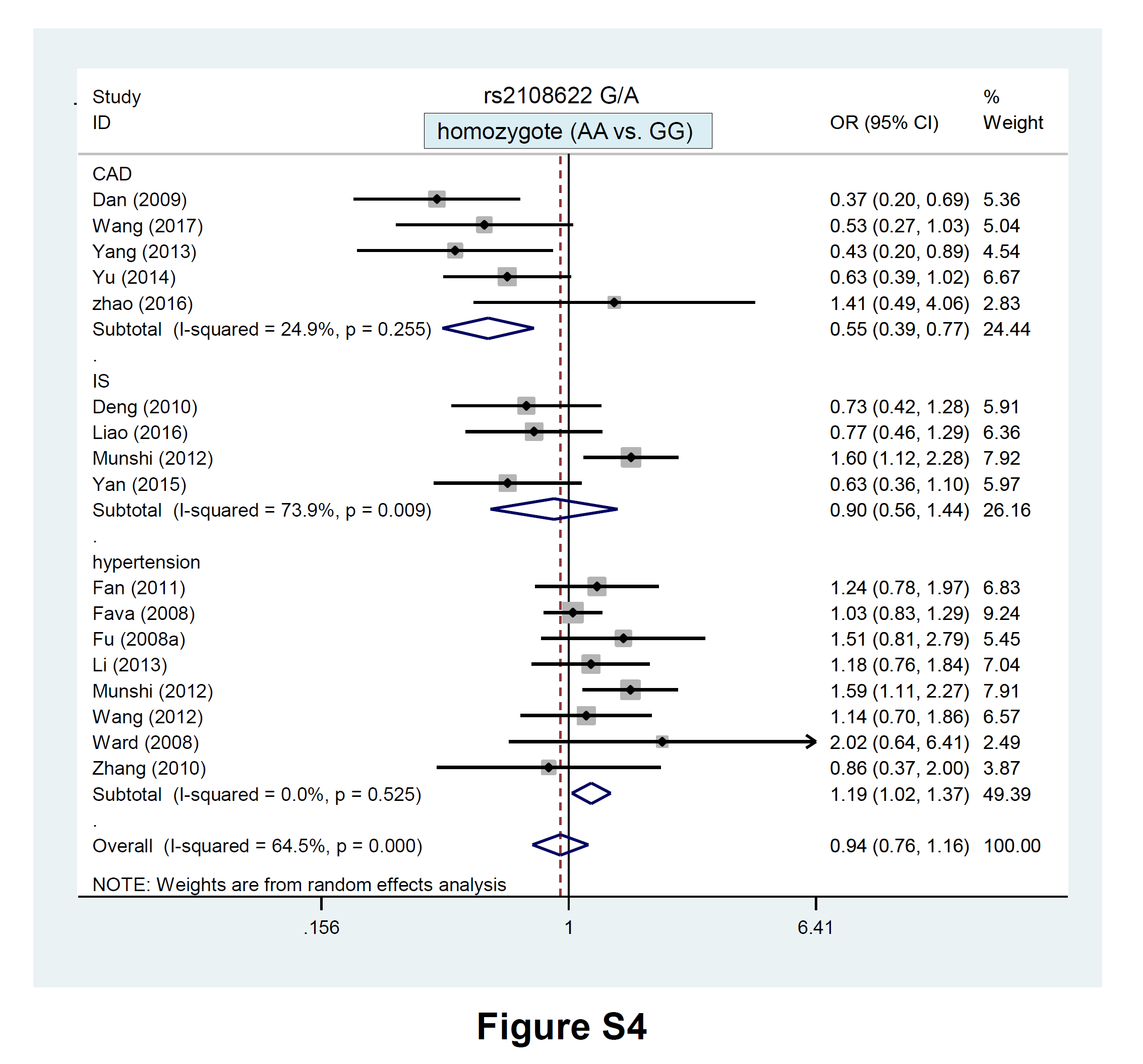

Supplement: Supplementary file 5 — Subgroup analysis by disease type of the association between the CYP4AF2 rs2108622 polymorphism and the risk of cardiovascular and cerebrovascular diseases under the homozygote (AA vs. GG) model. (TIFF 1760 kb) [file 12872_2018_763_MOESM5_ESM.tif]

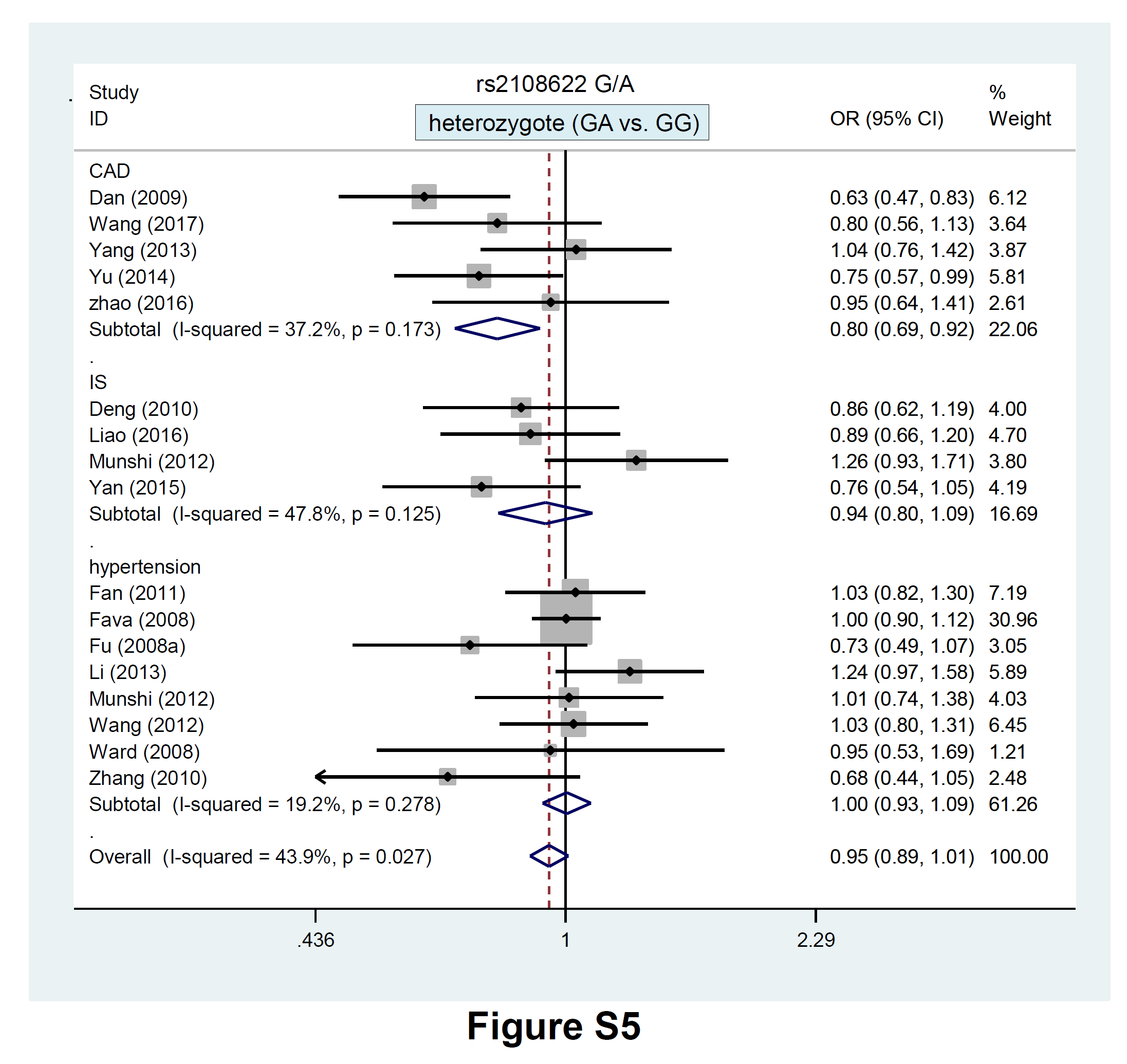

Supplement: Supplementary file 6 — Subgroup analysis by disease type of the association between the CYP4AF2 rs2108622 polymorphism and the risk of cardiovascular and cerebrovascular diseases under the heterozygote (GA vs. GG) model. (TIFF 1717 kb) [file 12872_2018_763_MOESM6_ESM.tif]

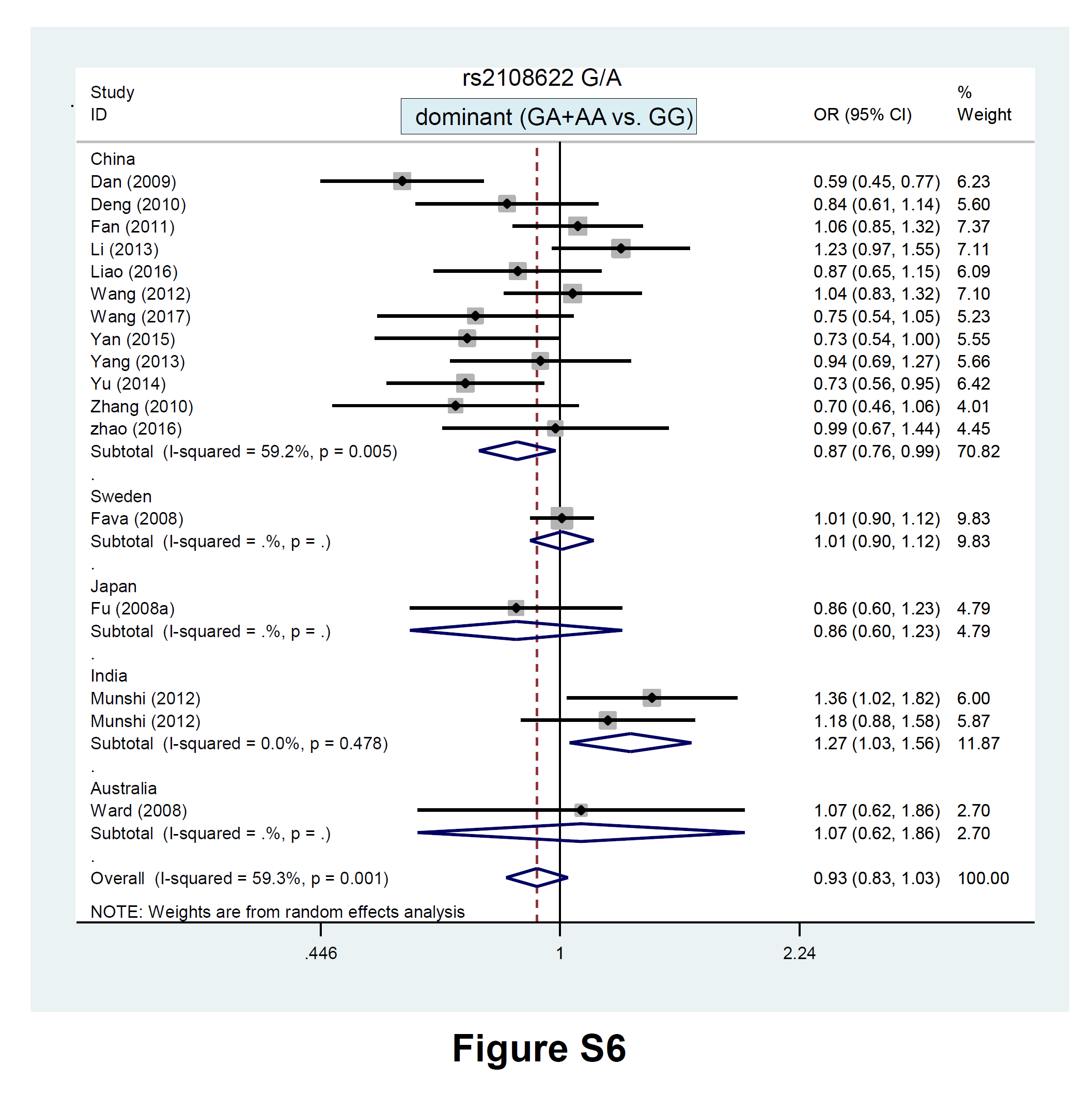

Supplement: Supplementary file 7 — Subgroup analysis by disease type of the association between the CYP4AF2 rs2108622 polymorphism and the risk of cardiovascular and cerebrovascular diseases under the dominant (GA + AA vs. GG) model. (TIFF 1689 kb) [file 12872_2018_763_MOESM7_ESM.tif]

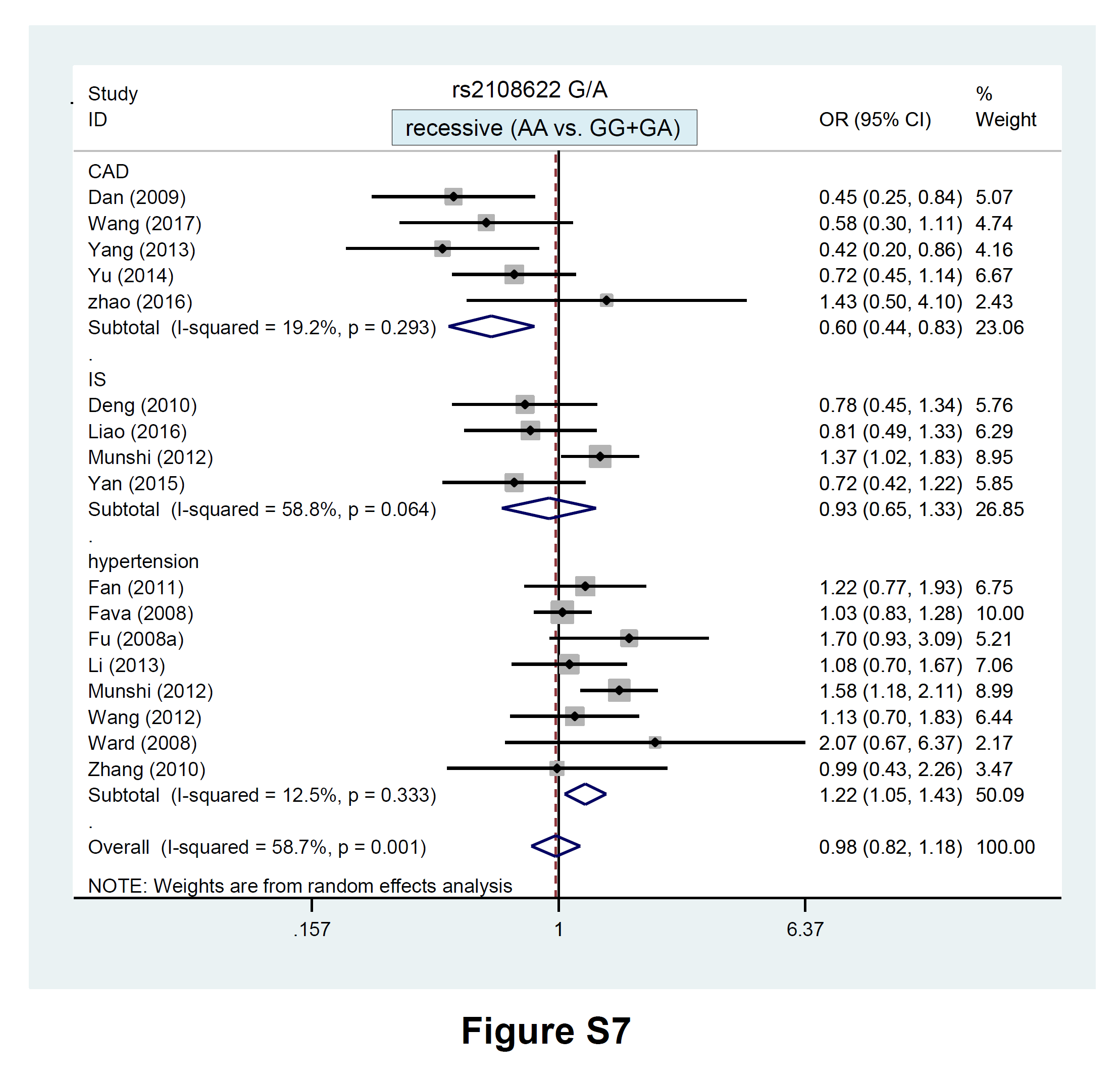

Supplement: Supplementary file 8 — Subgroup analysis by disease type of the association between the CYP4AF2 rs2108622 polymorphism and the risk of cardiovascular and cerebrovascular diseases under the recessive (AA vs. GG + GA) model. (TIFF 1792 kb) [file 12872_2018_763_MOESM8_ESM.tif]

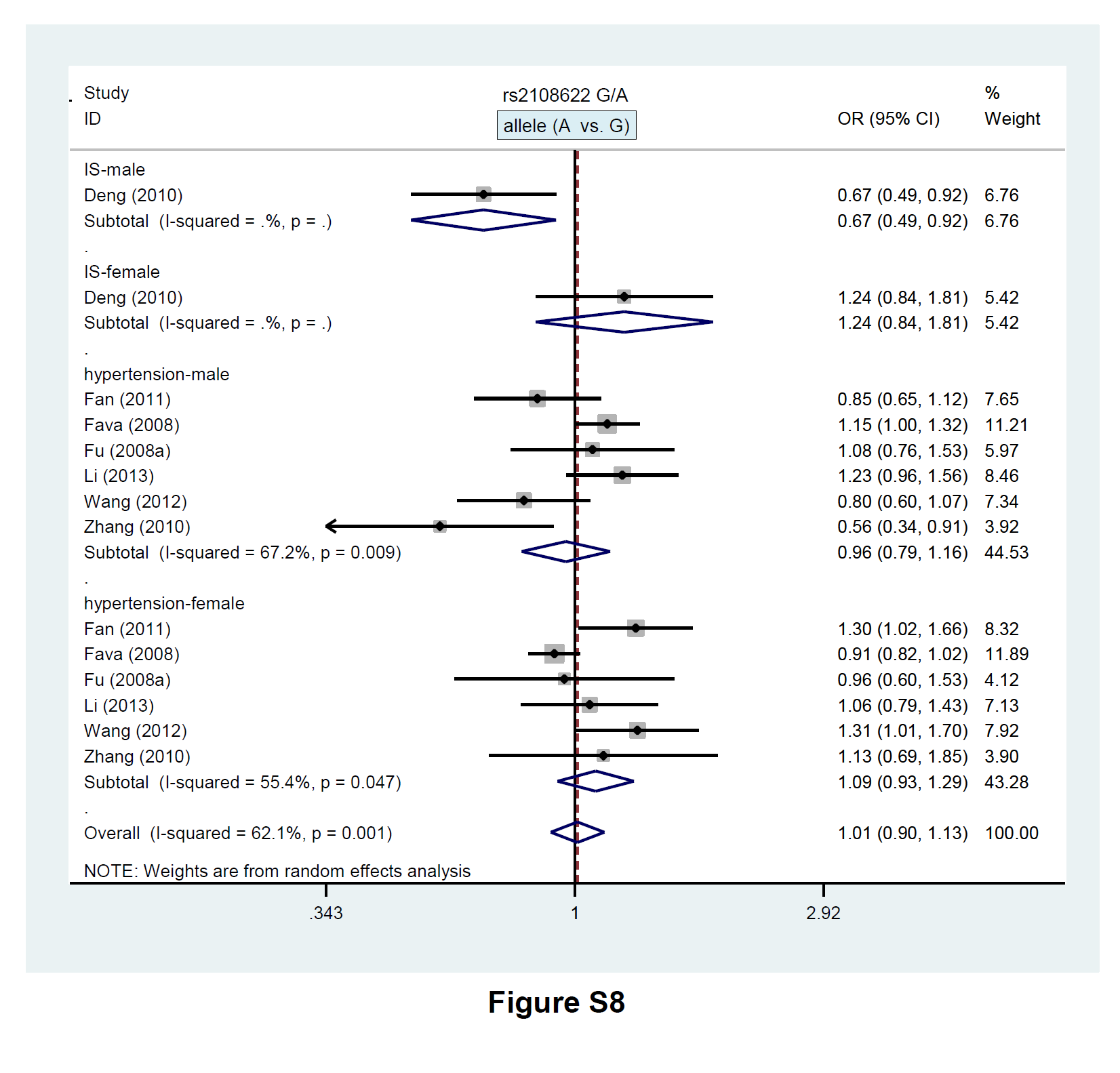

Supplement: Supplementary file 9 — Subgroup analysis by gender of the association between the CYP4AF2 rs2108622 polymorphism and the risk of cardiovascular and cerebrovascular diseases under the allele (A vs. G) model. (TIFF 1045 kb) [file 12872_2018_763_MOESM9_ESM.tif]
